# Attentional Blink Is Hierarchically Modulated by Phonological, Morphological, Semantic and Lexical Connections between Two Chinese Characters

**DOI:** 10.1371/journal.pone.0104626

**Published:** 2014-08-07

**Authors:** Hong-Wen Cao, Kai-Bin Jin, Chao-Yi Li, Hong-Mei Yan

**Affiliations:** 1 Key Laboratory for NeuroInformation of Ministry of Education, University of Electronic Science and Technology of China, Chengdu, China; 2 Shanghai Institutes of Biological Sciences, Chinese Academy of Sciences, Shanghai, China; University of Akron, United States of America

## Abstract

The ability to identify the second of two targets (T2) is impaired if that target is presented less than ∼500 ms after the first (T1). This transient deficit is known as attentional blink (AB). Previous studies have suggested that the magnitude of the AB effect can be modulated by manipulating the allocation of attentional resources to T1 or T2. However, few experiments have used Chinese characters and words to explore this phenomenon. The existence of lexical, semantic, phonological and morphological connections between Chinese characters has been well established, and understanding these connections may improve our knowledge of reading Chinese. In this study, we employed varying connections between T1 and T2 and examined how these connections modulate the AB effect. We found that the strongest AB was observed when the two Chinese characters were completely unrelated, while the AB was reduced when T1 and T2 were phonologically, orthographically or semantically related and was almost completely eliminated when T1 and T2 were united in a lexical phrase. The order of activation between Chinese characters was identified as follows: (a) lexical phrases, (b) semantic connection, (c) morphological connection, (d) phonological connection and (e) unrelated words.

## Introduction

Humans possess a remarkable ability to recognize a visual target even when it is embedded within a *rapid serial visual presentation* (RSVP) stream that includes spatially overlapping distractors. However, when two such targets (conventionally labeled T1 and T2) are presented successively, the ability to recognize T2 is severely impaired if it occurs within approximately 500 ms after T1. The identification of T2 is unimpaired after longer target separations or when the presentation of T1 is ignored [1], [2]. This transient deficit is known as attentional blink (AB) [2], which has been a major topic in attention research. The AB has not only been established as an effective means of studying the timing of attention and memory consolidation, but it has also provided researchers with a tool to examine one of the most interesting topics in cognitive neuroscience, namely, human consciousness.

Over the past two decades, several studies have established that the AB effect can be observed with a wide variety of stimuli, such as symbols [3], pictures [4], faces [5] or words [6]–[8]. Typically, the AB occurs during a RSVP stream, but it can also be obtained with auditory [9], [10] or tactile paradigms [11]. The AB effect is thought to reflect a very general property of perceptual awareness with broad implications for understanding how the brain perceives any task-relevant stimulus [12]. Several studies have suggested that the magnitude of the AB effect can be modulated by adding an extraneous cognitive load or by manipulating the allocation of attentional resources to T1 or T2. A stronger AB effect has been observed when the time required to process T1 is prolonged, such as when identification is more difficult [13]–[18]. In contrast, a weaker AB effect has been observed when the subjects are asked to listen to task-irrelevant music or think about their holiday during the experiment [19]. The magnitude of the AB is also attenuated if the target stimuli are associatively or semantically related pairs of words [20]. Our understanding of the AB has rapidly increased in recent years based on new neurophysiological evidence and computational accounts of attentional processes [12], [21]–[23]. However, several basic questions still remain unexplored. For example, while the relationship between T1 and T2 has been established as one of the key factors that modulates the AB effect, the role of the graphical or semantic connection of the two targets remains unclear. Specifically, both the effect of the low-level visual and high-level lexical characteristics on attention, memory, and consciousness at different stages of the AB, as well as the occurrence of perceptual priming of these characteristics in the forward or reverse direction, are not well understood.

Previous studies have primarily explored the AB effect with English alphabets, symbols, digits, pictures, etc.; however, few experiments have been conducted with Chinese characters and words. Chinese is a logographic language that differs markedly from alphabetic English in terms of its orthography, phonology, semantics and phrase structures. English words have a linear structure, whereas Chinese characters have a square, nonlinear configuration. The composition of Chinese characters contains spatial information, and stroke assembly rules dictate the location of each stroke that is then constructed into a square shape. The pronunciation of each character consists of an initial consonant, a vowel, sometimes a final consonant and a tone (first, second, third or fourth). Complicated connections exist between characters in the Chinese language, including morphological, phonological, semantic and lexical connections. Each of these connections plays a role in the recognition and identification of Chinese characters and may improve our understanding when reading. Several previous studies have addressed the crucial issue of the sequence of facilitation with conflicting results. For example, Perfetti and Tan proposed that graphic information is activated first, followed by phonological and then semantic information [24]. In contrast, Zhou et al. found that semantic information was activated at least as early and just as strongly as phonological information [25]. In a third study, Liu et al. used event-related potential to examine the processing of phonological, orthographical, and lexical information on Chinese characters in a sentence and concluded that the extraction of phonological information occurred earlier than that of orthographical and lexical information [26]. Finally, Tsai et al. investigated the modulation of preview duration by parafoveal information extraction and proposed that the orders of activation are identical, semantically related, phonologically related, orthographically related and unrelated [27].

Taken together, these studies suggest that the complex connections between two Chinese characters provide a good basis for examining the relationship between T1 and T2 in AB. Additionally, the AB paradigm may also inform us about how tightly related Chinese characters are.

In this study, we set out to answer the following three questions: (a) Does the AB effect occur during rapid serial Chinese character presentation? (b) How does the phonological, orthographical, semantic, and lexical information between two Chinese characters contribute to the modulation of the AB? (c) What is the order of facilitation of these low-level visual or high-level lexical characteristics in Chinese character recognition and identification? We used a classical AB experimental procedure in which pairs of Chinese characters in five different categories were presented as stimuli. Our results showed that a strong AB effect was obtained when T1 and T2 were unrelated. In contrast, gradual attenuation of the AB was observed with two homophonic, morphologically similar or semantic pairs. Finally, the AB effect was eliminated completely when T1 and T2 were united in a lexical phrase. We concluded that the order of activation of Chinese characteristics was (a) lexical phrases, (b) semantic connection, (c) morphological connection, (d) phonological connection and (e) unrelated words.

## Materials and Methods

### Participants

Twenty right-handed subjects (eleven females) aged 21–31 (average age 25.25) participated in these experiments. The subjects were all native Chinese speakers, although they all had some additional knowledge of spoken and written English. All subjects had normal or corrected-to-normal vision and provided written informed consent prior to participation. The experimental paradigms were approved by the Ethics and Human Participants in Research Committee at the University of Electronic Sciences and Technology of China in Chengdu, China. All subjects were blind with respect to the purpose of the experiments and were familiarized with the task by undergoing an initial training of 80 trials before the experimental phase began.

### Apparatus

The tasks were performed in a sound-attenuated room that was specially designed for psychophysics experiments, and the room illumination was held constant for all participants. The subjects viewed the display from a distance of 60 cm, and their head movements were restricted by forehead and chin rests. The stimuli appeared on the center of a grey background that was adjusted to a mean luminance of approximately 9.1 cd/m^2^. The stimulus presentation program was compiled by MATLAB (the MathWorks, US) using Psychtoolbox (Psychophysics Toolbox Version 3, US). The stimuli were presented on a display computer with a high resolution color monitor (1024×1280 pixels, 3×8 bit RGB) and a 100 Hz refresh rate.

### Stimuli

T1 and T2 consisted of paired Chinese characters in five categories: (1) homophonic characters, which are pronounced the same and are identical in tone but are unrelated in semantic meaning, component or origin, e.g., “案” and “岸” (both pronounced “AN”), (2) orthographically similar characters, which share an identical component and are structurally similar but differ in pronunciation and semantic meaning, e.g., “极” (pronounced “JI” and means “best”) and “吸” (pronounced “XI” and means “suck”); in this example, the two characters share visual similarity because they both contain “及”, (3) semantically related characters that are completely different in spelling, sound, component or origin, and cannot be united into a lexical phrase, but are somewhat related in semantic meaning, such as synonyms, antonyms and those pairs which belong to same kinds, e.g., “绿” (green) and “黄” (yellow), both semantically related to color, (4) lexical two-character Chinese phrases, e.g., “舒” (pronounced “SHU” and means “stretch”) and “服” (pronounced “FU” and means “dress”), which mean comfortable when they are written together; however, they are completely unrelated in terms of pronunciation and individual meaning, and (5) unrelated characters, which are completely dissimilar in graphic, phonological, semantic or lexical information between the pairs, e.g., “质” (pronounced “ZHI” and means “quality”) and “居” (pronounced “JU” and means “reside”).

The materials are often-used characters which are rather familiar to the readers. Each category included 150 stimulus pairs for a total of 750 pairs of Chinese characters. All chosen characters occur with high frequency in the Chinese language according to the Modern Chinese Frequency Dictionary [28], the general standard table of Chinese characters [29], and Frequency Dictionary of Modern Chinese words in common uses [30]. The mean frequencies of T1 and T2 are 80.65 (SD = 15.36) and 81.33 (SD = 13.54) per million for unrelated characters, 77.26 (SD = 18.66) and 78.19 (SD = 20.67) for homophonic, 81.70 (SD = 16.65) and 81.97 (SD = 15.82) for orthographically similar, 83.09 (SD = 8.88) and 81.93 (SD = 18.23) for semantically related, 75.29 (SD = 17.80) and 76.22 (SD = 22.78) for lexical pairs according to the Modern Chinese Frequency Dictionary [28], respectively. One-way analysis of variance (ANOVA) revealed no significant differences for the frequencies of T1 and T2 across all five conditions (p>0.05 in all cases). For the lexical condition, all the lexical two-character Chinese phrases are highly frequent ones according to the Modern Chinese Frequency Dictionary with mean frequency of 70.83 (SD = 10.19). The visual complexity of the characters was balanced across stimuli types, and each character was composed of six to thirteen strokes. The mean numbers of strokes of T1 and T2 are 7.87 (SD = 2.20) and 7.40 (SD = 2.38) for unrelated, 7.13 (SD = 1.46) and 7.20 (SD = 1.97) for homophonic, 7.87 (SD = 1.92) and 7.53 (SD = 1.99) for orthographically similar, 7.53 (SD = 2.83) and 7.40 (SD = 2.41) for semantically related, 7.20 (SD = 1.74) and 7.40 (SD = 2.26) for lexical characters, respectively. There are no significant differences for strokes among the five conditions (p>0.05 in all cases).

The orthographic, phonological, semantic and lexical connections of each chosen pair were pre-assessed by 10 subjects who did not participate in the experimental phase of this study. A seven-point scoring system was adopted with a score ranging from 1 (lowest connection) to 7 (highest connection). Four types of connection rating were carried out for each condition of T1–T2 pairs separately. Table 1 illustrated their mean rating scores and the standard deviations.

**Table 1:** Mean rating scores and the standard deviations of the five types of connections for the five conditions of T1–T2 pairs (M±SD).

Connection type				
T1–T2 Category	Homophonic	Orthographically similar	Semantically related	Lexical
**Unrelated pairs**	1.16±0.370	1.16±0.422	1.08±0.274	1.08±0.274
**Homophonic pairs**	**6.96±0.197**	1.06±0.239	1.01±0.100	1.02±0.200
**Orthographically similar pairs**	1.03±0.171	**6.95±0.219**	1.03±0.171	1.09±0.351
**Semantically related pairs**	1.00±0.000	1.01±0.100	**6.87±0.338**	1.16±0.395
**Lexical pairs**	1.04±0.197	1.04±0.197	1.21±0.409	**6.98±0.141**

From table 1, each condition of T1–T2 pairs has high rating scores about the corresponding connection the subjects rated but low about the other connections, which means each condition of T1–T2 pairs is closely related with its corresponding connection. Take homophonic pairs for example. All the homophonic pairs are rated to be highly homophonically connected but lowly connected in orthographic, semantic and lexical connections. For unrelated condition, all the T1–T2 pairs are rated to be lowly connected in any of the four connection types.

**Figure 1:**
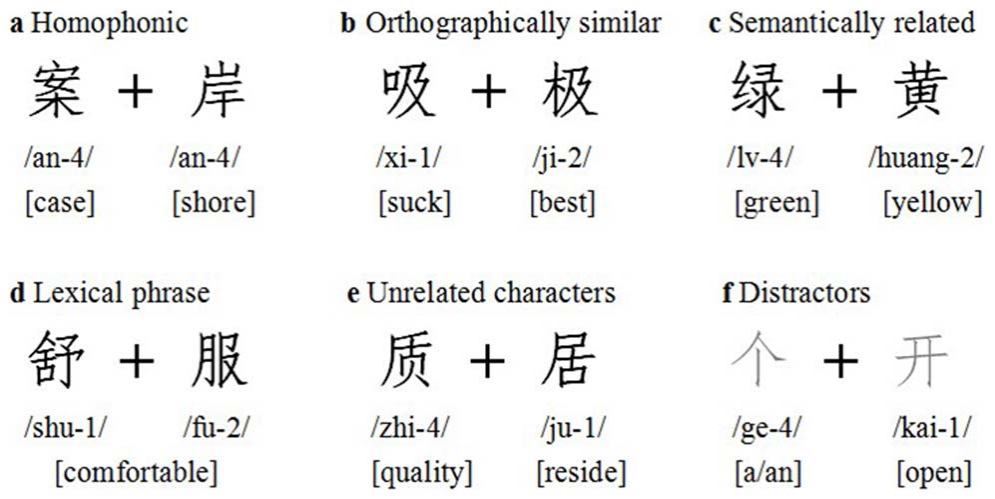
Examples of five experimental stimuli pairs and one distractor that fall into the following categories: (a) homophonic characters, (b) orthographically similar characters, (c) semantically related characters, (d) lexical two-character Chinese phrases, (e) unrelated characters, and (f) distractors. The pronunciation of each character according to the Chinese phonetic labeling system (i.e., pinyin) is listed below the character, the number at the end of the pronunciation denotes the tone, and the English meaning is listed at the bottom of each character within a square bracket.

The distractors consisted of 72 of the most commonly used simple Chinese characters, which were composed of two to seven strokes. These characters were unrelated in terms of their graphic, phonological, semantic and lexical information. Figure 1 contains an example from each of the five categories of experimental pairs as well as a distractor. The English translations are listed underneath the Chinese characters.

Each character was displayed on the screen at the same size (0.86°×0.95°). The stimuli pairs and distractors were randomly chosen for each trial. The characters chosen as T1 and T2 for the discrimination task were displayed in bold, while the distractors were presented in a normal font.

### Experimental Paradigm

**Figure 2:**
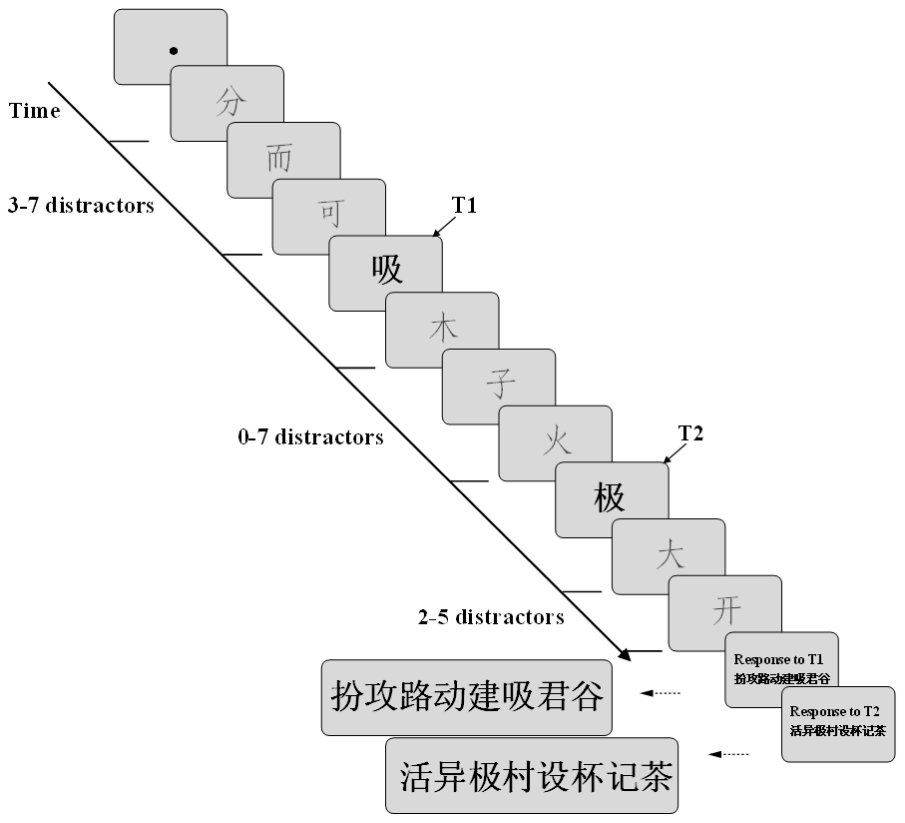
The sequence of events during the experimental paradigm for each trial. The presentation duration of characters was 60 msec/item. T1 and T2 were displayed in bold, while the distractors were presented in normal font. The subjects were asked to use a computer mouse to identify T1 and T2 from two different panels of eight Chinese characters.

Figure 2 shows the sequence of events during the experimental phase. Each trial started with the appearance of a black fixation dot (0.3° in diameter) at the center of the screen, which was extinguished after 1000 ms and was followed by the appearance of 3–7 distractors. Next, the two targets were selected randomly from the two stimulus corpora and presented in sequence but randomly separated by 0–7 distractors. Finally, 2–5 distractors were presented as a backward mask after the second target. The sequence of distractors varied randomly, but identical characters never appeared in a single trial. The presentation duration of each character was 60 msec/item. If there was no distractor between the two targets (i.e., T2 immediately followed T1), the stimulus onset asynchrony (SOA) was 60 msec, which is referred to as lag 1 [31]. Likewise, when the two targets were separated by 1–7 distractors, the SOA was designated as lag 2–8, respectively. After the RSVP, the first panel containing eight bold, black Chinese characters was shown on the screen, and the subjects were asked to use a computer mouse to identify T1. After T1 was chosen, a second panel with another eight characters was presented, and the subjects were asked to identify T2. The eight unique characters in the panel were randomly selected from a set of Chinese characters. Once the subjects made their choices, the next trial began. After the subjects’ identification of T1 and T2 was recorded, there was no feedback to the participants about their choice. Each subject performed in two sessions, and each session included 15 blocks of 25 trials resulting in a total of 750 individual trials.

The control paradigm utilized a similar setup, but the subjects were asked to identify T2 only. Similarly, the same number of trials was carried on for each subject in the single-task condition.

## Results

### Identification of T1 and T2 in the dual-task paradigm

**Figure 3:**
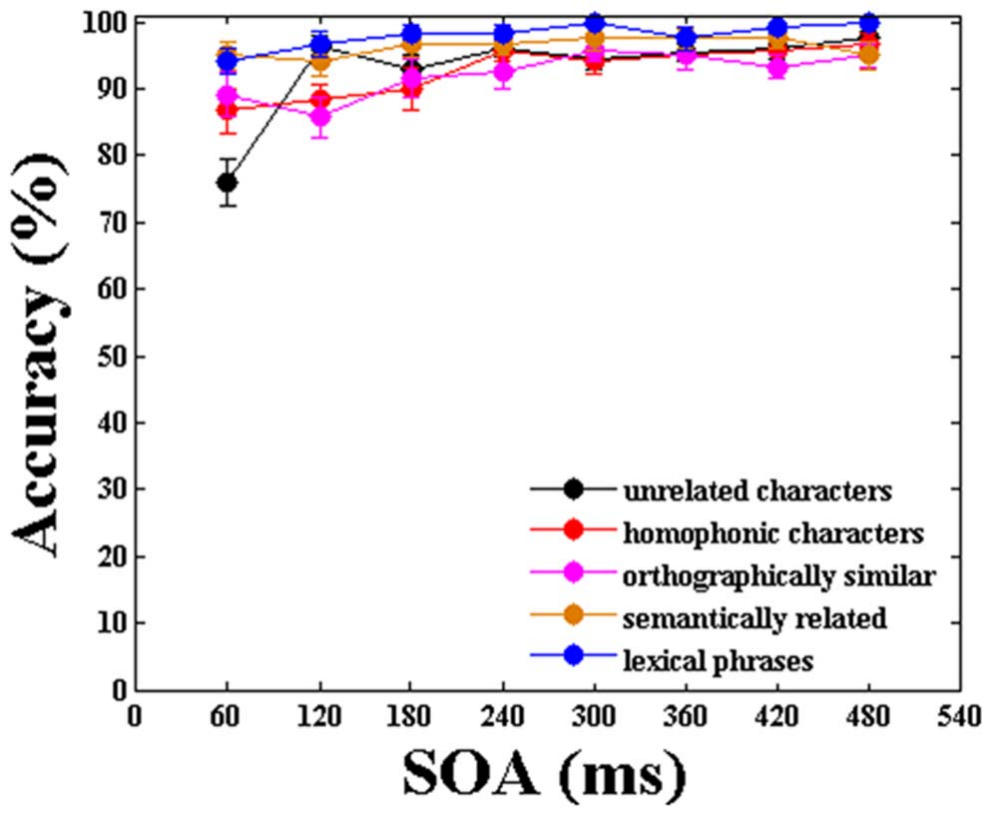
Mean accuracy in identifying T1 at all SOAs in five stimulus categories during dual-task paradigm. The error bars represent the standard error.

Figure 3 shows the accuracy with which the subjects could identify T1 at all SOAs in each of the five stimulus categories. The mean performance under all five conditions was above 90%. A two-variable (Temporal lag × Category) repeated measures ANOVA revealed that there were no significant differences among the categories across SOAs in a subject analysis (F1(4,95) = 1.396, p = 0.241) and an item analysis (F2(4,745) = 1.371, p = 0.243).

**Figure 4:**
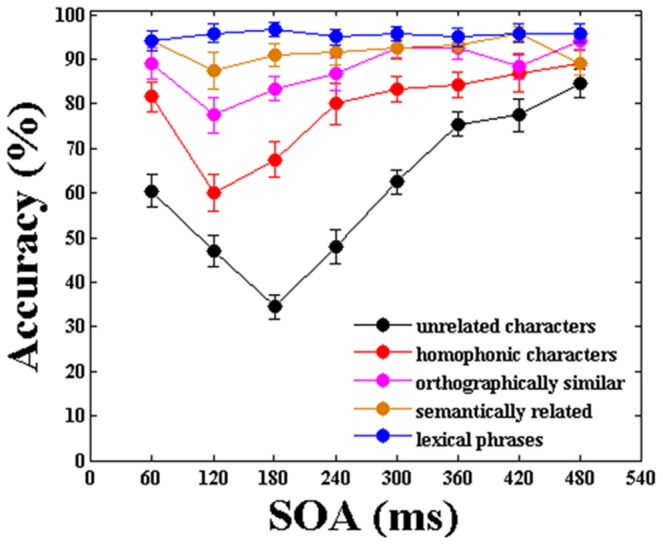
Mean accuracy of T2 for trials in which T1 was detected correctly in five categories. The error bars indicate the standard error.

Figure 4 indicates the accuracy with which the subjects were able to identify T2 at all SOAs for the trials in which T1 was correctly recognized in all five categories. The average performances in identifying T2 when T1 had been correctly detected under unrelated, phonological, morphological, semantic and lexical conditions was 61.25%, 79.08%, 88.02%, 91.87% and 95.52%, respectively. First, let us have a general look at Figure 4. A strong AB effect was observed when T1 and T2 were two unrelated Chinese characters. The subjects had more difficulty identifying T2 when that stimulus was presented in close temporal proximity to T1. However, gradual attenuation of the AB was observed when T1 and T2 were two phonologically, morphologically or semantically related characters. Namely, the performance of T2 was somewhat impaired under certain lag period under these three conditions, but its amplitude became gradually weaker compared with the unrelated category. Furthermore, the AB effect disappeared completely when T1 and T2 were united in a lexical, two-character Chinese phrase. The subjects’ accuracy in identifying T2 was almost identical for all temporal lags under lexical condition.

Following, we present the statistical results. A two-way ANOVA (factors: Temporal lag × Category) was carried out among the categories across SOAs. A main effect of lag was obtained (F1(7,152) = 9.245, p = 0.000; F2(4,1192) = 10.551, p = 0.000). A main effect of condition was also found (F1(4,95) = 17.263, p = 0.000; F2(4,745) = 31.114, p = 0.000). The results revealed that subjects’ discrimination of T2 varied significantly for both category conditions and temporal lags. Besides, ANOVA showed a significant condition × lag interaction (F1(28,665) = 2.391, p = 0.000; F2(28,5215) = 2.569, p = 0.000). Post-hoc multiple comparison test revealed striking differences between the homophonic, orthographic, semantic, and lexical categories compared to the unrelated category (p = 0.001, 0.000, 0.000 and 0.000, respectively). Additionally, the subjects’ performance in the homophonic category was significantly different from their performance in the semantic and lexical categories (p = 0.010 and 0.001, respectively).

One-way ANOVAs (factor: Temporal lag) were also carried out for each of the five conditions to reveal the details of the differences. For unrelated condition, ANOVA revealed a significant effect of temporal lag (F1(7,152) = 28.315, p = 0.000; F2(7,1192) = 16.735, p = 0.000). This attentional deficit was strongest at lag 3 (approximately 180 ms), then the performance of T2 improved with increasing lag. The result was generally consistent with the findings reported by Raymond et al. [2]. Post-hoc multiple comparison test revealed significant differences between lags 1–5 and lag 8 (lag 1 (p = 0.000), 2 (p = 0.000), 3 (p = 0.000), 4 (p = 0.000), 5(p = 0.002), two-tailed compared to lag 8 individually), while there were no significant differences between lags 6–7 and lag 8 (p = 0.053 and 0.131, respectively). One-way ANOVAs also showed AB patterns in homophonic (F1(7,152) = 7.517, p = 0.000; F2(7,1192) = 8.124, p = 0.000) and orthographically similar conditions (F1(7,152) = 3.448, p = 0.002; F2(7,1192) = 3.579, p = 0.001). The performance of T2|T1 at lag 1 was significantly higher than that at lag 2 (p = 0.000 in homophonic and p = 0.040 in orthographical) and lag 3 (p = 0.009 in homophonic). The attentional deficit was strongest at lag 2, and performances recovered to the asymptote more quickly (i.e., the duration of the AB became gradually shorter). The performance of T2|T1 at lag 2 and lag3 was significantly lower than that at lag 5 (p = 0.000, p = 0.000 in homophonic and p = 0.002, p = 0.008 in morphological). Although ANOVA showed there was no effect of lag in the semantically related condition (F1(7,152) = 1.046, p = 0.402; F2(7,1192) = 1.202, p = 0.299), but there was a significant difference between lag 2 and lag 7 (p = 0.038). However, ANOVA showed there was no significant effect of lag in lexical condition (F1(7,152) = 0.155, p = 0.993; F2(7,1192) = 0.164, p = 0.989). The results revealed that T2|T1 performance was sensitive to the relatedness of T1 and T2, especially at the shorter lags.

In conclusion, both Figure 4 and the statistical results indicate that AB is hierarchically modulated by phonological, morphological, semantic and lexical connections between two Chinese characters.

### Identification of T2 in the single-task paradigm

**Figure 5:**
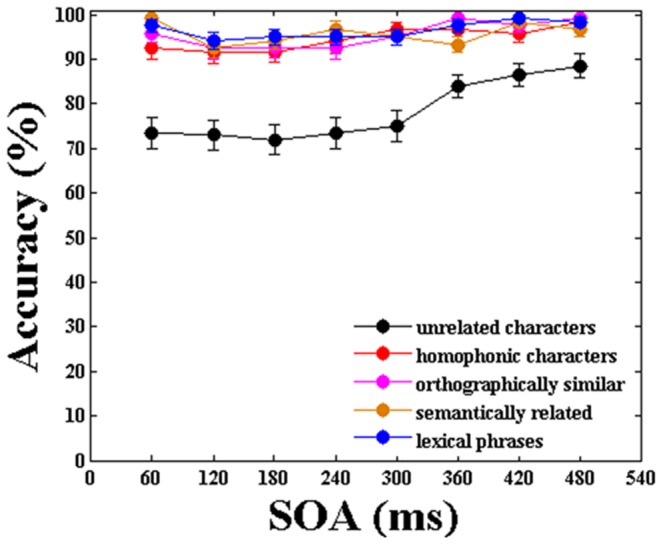
Mean accuracy in identifying T2 in the single-task experiment. The error bars represent the standard error.

Figure 5 shows the subjects’ mean accuracy in identifying T2 during the single-task experiment at all SOAs in each of the five categories. The mean performance in identifying T2 under unrelated, phonological, morphological, semantic and phrase conditions were 78.25%, 94.69%, 95.52%, 95.72%, and 96.46%, respectively. A two-way ANOVA showed that there was no significant main effect of temporal lag (F1(7,152) = 1.903, p = 0.116; F2(7,1192) = 1.389, p = 0.236). The AB effect was absent in the single-task experiment, which is consistent with previous reports indicating that awareness of T1 is necessary for its occurrence.

Additionally, we also found that the subjects’ accuracy in identifying T2 in unrelated stimulus pairs was the lowest of all categories. The ANOVA revealed a significant main effect of category (F1(4,95) = 58.010, p = 0.000; F2(4,745) = 59.180, p = 0.000). A post-hoc multiple comparison test revealed a significant difference when the subjects’ performance in the unrelated category was compared to their performance in the other four categories (p = 0.000 in all cases). However, no significant difference was observed among the other four conditions (p>0.05).

## Discussion

The purpose of this study was to employ Chinese characters as stimuli to investigate the effect of their relationship in modulating the AB effect. The results indicated that a strong AB was present when T1 and T2 were two completely unrelated Chinese characters. However, when a connection exists between the two characters, the magnitude and duration of the AB was modulated. A gradual attenuation of the AB was detected with two homophonically, morphologically or semantically related Chinese characters. Finally, the AB effect disappeared when T1 and T2 were united in a lexical phrase. In the experiment, T1 was presented randomly, and T2 could not be predicted by T1 in advance. However, the relationship of T1/T2 could hierarchically modulate the discrimination of T2 in the rapid serial visual presentation.

One question addressed here is why the AB can be attenuated by two related Chinese characters. There is an ongoing and longstanding debate about the underlying cause of the AB because it is not known whether the blink reflects limited processing resources or is the product of attentional control. The limited-capacity model assumes that the AB occurs because limited attentional resources are allocated to the leading target at the expense of the trailing target [32], [33]. Similar assumptions underlie the two-stage model of Chun and Potter [3], in which the AB deficit is said to occur when the second target arrives while high-level resources are occupied with the first target. However, Di Lollo et al. proposed the hypothesis that the AB arises from a temporary loss of control over the prevailing attentional set. This lapse in control renders the observer vulnerable to an exogenously triggered switch in the attentional set. If T2 belongs to the same category as or is similar to T1, it will match the current configuration of the system and will gain access to further processing [34]. Our results strongly support the latter hypothesis. When T1 and T2 consisted of completely unrelated Chinese characters in the dual-task paradigm, the brain was initially configured to optimize its performance on the first target, which was therefore processed quickly and accurately. A temporary loss of control over the prevailing attentional set resulted in a strong AB effect. However, when T2 shared certain phonological, morphological or semantic similarity with T1, the priming effect led to the redistribution or accelerated allocation of attention, which resulted in an improved performance in identifying T2. As the targets became more related, attention was redistributed faster and the AB became smaller. The AB completely disappeared when the targets were united in lexical phrases because these fixed phrases were usually presented and processed as a whole. This attenuation in AB was consistent with a previous study by Potter et al. that involved semantic priming with English words [20].

A second question addressed in this study is whether the priming of perceptual or semantic characteristics functionally occurs in a forward or reverse direction. Potter et al. [20] studied the time course of semantic priming between two associated English and Italian words and found that the priming occurred primarily in a functionally forward direction (i.e., from an identified T1 to an unidentified T2). However, they proposed that backward priming also occurred rapidly and immediately if T2 was the first word to be identified and T1 was close to the point of identification. In this case, the ability to identify T1 would be improved after seeing T2 if the targets shared semantic features. Our findings support the idea that the forward priming of phonological, morphological, semantic and lexical information indeed facilities the identification of T2 and results in the attenuation or elimination of the AB. However, the lack of any significant difference in the ability to identify T1 in response to unrelated stimuli compared to stimuli from any of the other categories at all SOAs suggests that there was no backward priming effect in our study. In the control experiment, the subjects were significantly more accurate in identifying T2 among stimuli from the four related categories than from the unrelated category. Although the subjects were instructed to only respond to T2 and ignore T1 in the single-task paradigm, the presence of T1 still might either interfere with the perception of an unrelated T2 or facilitate the identification of a related T2 in the RSVP stream.

Our observation of an attenuated hierarchy of the AB effect also suggests a specific order of prime facilitation during rapid recognition of related Chinese characters and the establishment of an identification stream. The order of activation appears to be arranged as follows: (a) lexical phrases, (b) semantic connection, (c) morphological connection, (d) phonological connection and (e) unrelated words. Several pieces of evidence support this conclusion. First, the importance of fixed phrases has been supported by many previous studies [35]. Our results also demonstrate that a united, lexical two-character phrase may form a holistic entry in the mental lexicon, which destroys the AB phenomenon. Second, the ability to comprehend the semantic meaning of words is the primary goal with any language, and semantic extraction is a high-level cognitive activity. We believe that high-level semantic similarity or activation is more powerful than low-level graphic or phonological similarity in the identification of Chinese characters. Third, Chinese is a logographic writing system in which morphologically similar characters share an identical component, which presents a more salient visual feature than monophonic pairs in visual paradigms. Finally, our findings were consistent with the theory by Tan et al. that phonological characteristics also play a role in Chinese word identification [36]; however, we found that this type of activation was quite weak overall and was only stronger compared to the unrelated condition.
